# Effect of Sc Microalloying on Interfacial Microstructural Evolution in Al/Mg Bimetallic Composites Fabricated by Lost Foam Compound Casting

**DOI:** 10.3390/ma19214443

**Published:** 2026-10-22

**Authors:** Jinxin Liu, Taoxiong Guo, Ziwei Peng, Xiaopeng Yu, Lihui Zhang, Xingyu Huang, Wenming Jiang

**Affiliations:** State Key Laboratory of Materials Processing and Die & Mould Technology, School of Materials Science and Engineering, Huazhong University of Science and Technology, Wuhan 430074, Chinam202671030@hust.edu.cn (L.Z.); m202671029@hust.edu.cn (X.H.)

**Keywords:** Al/Mg bimetallic composite, lost foam compound casting, Sc microalloying, interfacial microstructure, Mg_2_Si, Vickers hardness

## Abstract

Sc microalloying was introduced into A356 Al inserts to regulate interfacial microstructural evolution in A356/AZ91D bimetallic composites fabricated by lost foam compound casting. Nominal Sc additions of 0, 0.6, 0.9, and 1.2 wt.% were investigated using scanning electron microscopy, energy-dispersive spectroscopy, electron backscatter diffraction, elemental mapping, microstructural size statistics, and Vickers hardness testing. The results showed that Sc addition markedly reduced the interfacial reaction region. The total interface thickness decreased from 1750 μm in the Sc-free specimen to 1069, 1088, and 922 μm at 0.6, 0.9, and 1.2 wt.% Sc, respectively. Among the Sc-containing specimens, 0.6 wt.% Sc produced the most pronounced microstructural refinement, reducing the mean equivalent circular diameter of indexed interfacial microstructural units from 23 to 19 μm and that of Mg_2_Si from 17 to 14 μm while decreasing the amount of coarse γ-Al_12_Mg_17_ dendrites. EBSD revealed no marked change in the major reliably indexed phases or overall preferred orientation after Sc addition, whereas elemental mapping showed discrete Sc-enriched regions rather than a continuous Sc-rich interfacial layer. These results indicate that Al-side Sc microalloying regulates the interface mainly by restricting the expansion of the interfacial reaction region and modifying local solidification and second-phase growth, thereby promoting interface thinning and microstructural refinement.

## 1. Introduction

Aluminum (Al) and magnesium (Mg) are widely used lightweight metallic materials because of their low density, high specific strength, and favorable processing characteristics. However, molten magnesium alloys exhibit high chemical reactivity toward oxygen, making appropriate melt protection essential during casting. Reviews of Mg/Al compound casting have indicated that Al/Mg bimetals can combine the complementary advantages of these two lightweight alloys and therefore have considerable potential for transportation, aerospace, and other lightweight structural applications [1]. Recent studies on pulsed-current-assisted rolling of Mg/Al laminates and hot spinning of Mg/Al composite tubes have further shown that interfacial nanocrystalline structures, their evolution during subsequent annealing, and bonding strength are closely related [2,3]. Therefore, the interfacial microstructural state is a key factor governing the overall performance of Al/Mg bimetals.

Al/Mg bimetals can be fabricated through various dissimilar joining and composite-processing routes, including transient liquid-phase bonding with multiple interlayers, ultrasonically assisted soldering, friction stir additive manufacturing, magnetic pulse welding, compound casting, and solid–liquid cast rolling [4,5,6,7,8,9]. Among these methods, compound casting is relatively simple, provides high forming efficiency, and can directly produce a metallurgically bonded interface, making it suitable for the integrated fabrication of complex structures [8]. From a phase-equilibrium perspective, experimental investigations, thermodynamic assessments, and phase-diagram calculations of the Al–Mg–Zn and Al–Mg systems provide a fundamental basis for understanding the formation of reaction phases such as Al_3_Mg_2_ and Al_12_Mg_17_, whereas studies of the Al–Mg–Si system establish the phase-equilibrium conditions relevant to Mg_2_Si formation [10,11,12,13]. The ASM alloy phase-diagram handbook and binary alloy phase-diagram data provide supplementary references for interfacial phase identification [14,15]. In addition, indentation studies of dissimilar Al/Mg interfaces have demonstrated pronounced local differences in mechanical properties between the interfacial reaction zone and the two substrates [16]. Accordingly, suppressing excessive interfacial reactions and controlling the spatial scale of brittle reaction phases are important considerations in the design of Al/Mg bimetallic interfaces.

Microalloying and interlayer design are important approaches for regulating Al/Mg interfaces. Previous compound-casting studies have shown that Ce, La, Gd, and Nd can modify interfacial reactions, microstructures, and bonding or fracture behavior, although the specific responses induced by these elements differ [17,18,19,20]. The principal mechanisms and strategies of interlayer regulation in Mg/Al laminates have also been systematically summarized [21]. In addition, combining rare-earth alloying with ultrasonic vibration can further alter interfacial microstructures, strengthening mechanisms, and fracture behavior, and studies involving Ce, La, Gd, and Y have demonstrated pronounced coupling effects between the added elements and processing conditions [22,23,24,25]. Therefore, the effects of rare-earth or other microalloying elements on the interface should not be generalized simply as either “thickening” or “thinning”; instead, they should be evaluated in terms of the location of element introduction, reaction-phase constitution, and solidification conditions.

External fields and thermomechanical processing can also significantly regulate Al/Mg interfaces. Experimental and simulation studies of ultrasonic-vibration-assisted compound casting at different ultrasonic powers have shown that the processing parameters can markedly affect interfacial microstructural evolution and bonding properties [26,27]. In friction stir welding, Sn soldering, and the fabrication of Mg/Al composite plates, ultrasonic treatment and Ni or Ni-Al_2_O_3_ coatings have also been used to regulate or suppress the formation and growth of interfacial intermetallic compounds [28,29,30,31]. In addition, multi-pass friction stir processing combined with rolling, embedded rolling, and combinations of metallic interlayers with ultrasonic vibration can modify the interfacial microstructure and defect characteristics of composite plates or welded joints [32,33,34]. These studies indicate that external fields, plastic deformation, and interlayers can regulate interfacial evolution by altering mass transfer, heat input, and local reaction conditions.

Coatings, surface treatments, and transition foils constitute another class of strategies for interfacial regulation. High-velocity oxygen-fuel (HVOF)-sprayed Ni coatings, FeCoNiCrCu/Ni composite coatings, and high-entropy-alloy coatings combined with ultrasonic treatment have all been reported to improve Al/Mg interfacial bonding by altering local interfacial chemistry, second-phase distributions, or nanocrystalline microstructures [35,36,37]. Cyclic hot pressing with a Zn interlayer, Al surface pretreatment, a Mo coating combined with an ultrasonic field, and Zn/Ti transition foils have likewise been shown to regulate interfacial microstructures, reaction phases, and mechanical properties [38,39,40,41]. Overall, existing interface-control strategies mainly regulate reaction-layer development by modifying local composition, mass-transfer pathways, thermodynamic and kinetic conditions, and nucleation and growth behavior.

Scandium (Sc) is a commonly used microalloying element in aluminum alloys. Studies of A356 and A356.2 alloys have shown that Sc addition can affect the solidification microstructure, mechanical properties, and microstructural stability during thermal exposure [42,43]. Further studies of the Al-Sc system and Sc/Zr microalloying have demonstrated that Sc can form Al_3_Sc with an L1_2_-ordered structure and can contribute to grain refinement, precipitation strengthening, and thermal stability [44,45,46]. Multiscale microstructural characterization has revealed the nucleation and three-dimensional structure of Al_3_Sc, as well as the crystallographic orientation relationship at the Al_3_Sc/Al interface [47,48]. Solidification studies of dilute Al-Sc alloys have also shown that Al_3_Sc formation is closely related to the solidification conditions and that the cooling rate can markedly alter the morphology of primary Al_3_Sc particles [49,50].

From the perspective of second-phase stability, stable dispersoids are important for the high-temperature microstructural stability of castable aluminum alloys, and the loss of coherency, coarsening behavior, and nanoscale structural evolution of Al_3_Sc precipitates have been systematically investigated [51,52,53]. More specifically, studies on hypoeutectic Al–Mg–Si/Al–Mg_2_Si alloys have shown that Sc can simultaneously refine primary α-Al and modify eutectic Mg_2_Si; the proposed mechanisms include Al_3_Sc-assisted heterogeneous nucleation and, for Mg_2_Si, Sc segregation with associated constitutional undercooling at the growth front [54,55]. These findings provide mechanistic context for the present study but are not taken as direct evidence that the same nucleation pathway operates at the Al/Mg compound-cast interface. In addition, studies of primary Sc-related particles in high-Sc Al–Mg alloys and microstructural optimization in A356-Sc-Sr alloys indicate that Sc content and melt treatment can significantly affect as-cast microstructural evolution [56,57]. Unlike direct addition of a regulating element to the Mg melt, introducing Sc into the Al insert before compound casting may first modify the microstructure and local chemical state on the Al side and subsequently influence the Al/Mg interfacial reaction indirectly. Nevertheless, the interface thickness, microstructural morphology, and elemental distribution associated with this route of Sc introduction have not yet been systematically investigated.

The aim of this study was to evaluate how Sc microalloying of A356 Al inserts affects the interfacial reaction-zone thickness, phase constitution, microstructural characteristics, and local hardness distribution in A356/AZ91D bimetallic composites fabricated by lost foam compound casting. Unlike approaches in which a regulating element is added directly to the Mg melt, pre-alloying Sc into the A356 insert modifies the Al side before contact with the AZ91D melt. This route raises a specific mechanistic question: whether Sc can confine growth of the interfacial reaction region and modify subsequent interfacial solidification without changing the dominant Al–Mg reaction-phase system. Accordingly, A356/AZ91D bimetallic composites with nominal Sc additions of 0, 0.6, 0.9, and 1.2 wt.% in the Al insert were fabricated by lost foam compound casting. SEM/EDS, EBSD, elemental mapping, interface-thickness measurements, equivalent-circular-diameter statistics, and hardness testing were combined to distinguish changes in reaction-layer growth from changes in local solidification morphology. Particular attention was paid to whether Sc forms a continuous interfacial barrier, whether the major reaction phases change, and how Sc affects coarse γ-Al_12_Mg_17_ and Mg_2_Si. On this basis, a mechanism for Al-side Sc-mediated interfacial regulation is proposed.

## 2. Materials and Methods

### 2.1. Material Preparation

As-cast A356 aluminum alloy and AZ91D magnesium alloy were used as the solid insert and liquid matrix materials, respectively, for the Al/Mg bimetallic composites. The chemical compositions of the alloys were analyzed by inductively coupled plasma optical emission spectroscopy (ICP-OES), and the results are presented in Table 1. Commercial-grade A356 aluminum alloy, AZ91D magnesium alloy, and Al–2Sc master alloy were used as the starting materials. The measured compositions of the A356 and AZ91D ingots are reported in Table 1. The Al–2Sc master alloy had the following measured composition (wt.%): Sc 2.00, Fe 0.08, Si 0.06, Ca 0.01, and balance Al.

The A356 alloy mainly consisted of an *α*-Al matrix and eutectic Si. The A356 aluminum alloy was melted in a resistance furnace under a mixed CO_2_ and SF_6_ protective atmosphere. When the melt temperature reached 750 °C, an Al-2Sc master alloy was added, followed by mechanical stirring to promote Sc distribution and thereby produce A356-xSc alloys with different nominal Sc contents. After solidification, the Sc-free A356 alloy and the alloys with different Sc contents were machined into cylindrical inserts and designated as Group A, S1 (0.6 wt.% Sc), S2 (0.9 wt.% Sc), and S3 (1.2 wt.% Sc), respectively. The inserts were wire-cut to a diameter of 10 mm and a height of 129 mm. Their surfaces were sequentially ground with SiC abrasive papers up to 2000 grit, cleaned for 20 s in a 10 g/L NaOH solution, and then treated for 20 s in a mixed solution of 50% HNO_3_ + 50% HF. The inserts were subsequently ultrasonically cleaned in anhydrous ethanol for 10 min to remove residual surface contaminants. The treated A356 inserts were assembled with expanded polystyrene (EPS; density, 12 kg/m^3^) foam patterns, and a water-based refractory coating was uniformly applied to the surfaces of the EPS patterns.

**Figure 1:**
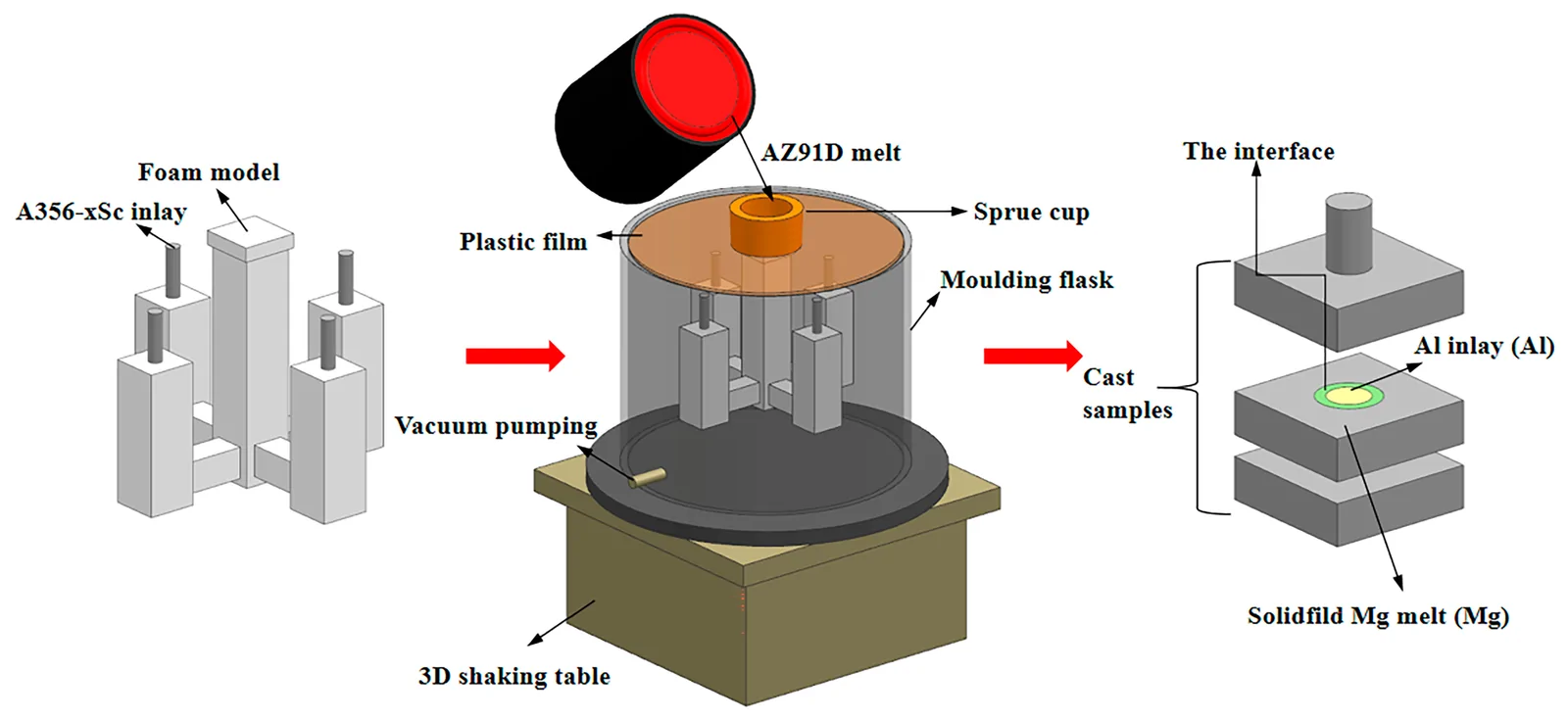
Schematic illustration of the lost foam compound casting process used to fabricate the Al/Mg bimetallic composites.

The AZ91D magnesium alloy ingots were melted in a resistance furnace under a CO_2_/SF_6_ protective atmosphere and held at 750 °C before pouring. As illustrated in Figure 1, the prepared Al alloy inserts were inserted into EPS foam patterns. A layer of refractory coating was applied to the pattern surfaces and allowed to dry naturally. The dried patterns were then fixed in a molding flask, and unbonded dry sand was added under vibration until the flask was filled, leaving the pouring cup exposed. Before pouring, vacuum suction was applied to the sand mold and maintained throughout pouring. The AZ91D melt was then poured into the evacuated mold at 750 °C. The CO_2_/SF_6_ gas mixture was used for melt protection during melting, whereas vacuum suction was used during pouring. Following cooling, the castings were removed from the sand mold and sectioned using a cutting machine to obtain characterization specimens. The recorded vacuum level during pouring was 0.03 MPa; vacuum suction was applied to promote mold filling and reduce casting defects. After cooling to room temperature, four groups of specimens were obtained: the A356/AZ91D bimetallic composite without Sc (Group A) and the specimens containing 0.6, 0.9, and 1.2 wt.% Sc (Groups S1, S2, and S3, respectively).

### 2.2. Microstructural Characterization

The interfacial microstructures of the Al/Mg bimetallic composites were examined by scanning electron microscopy (SEM) coupled with energy-dispersive X-ray spectroscopy (EDS). SEM specimens were sectioned perpendicular to the Al/Mg interface and mechanically ground and polished to obtain flat cross-sections.

A ZEISS EVO 15 environmental scanning electron microscope (ESEM; Carl Zeiss Microscopy GmbH, Jena, Germany)operated at an accelerating voltage of 20 kV and equipped for EDS point analysis and elemental mapping was used to determine the local elemental compositions and spatial distributions in the major interfacial microstructural regions. The interface thickness was measured at multiple representative positions along the interface normal using low-magnification SEM images. The thicknesses of the intermetallic compound (IMC) layer and the eutectic layer were measured separately, and their sum was taken as the total interface thickness. Phase assignments based on the EDS point analyses were inferred by comparing measured elemental compositions with theoretical stoichiometric ratios, relevant phase diagrams, and SEM morphologies. Because EDS provides elemental rather than crystallographic information, these assignments were regarded as composition-based interpretations rather than independent crystal-structure confirmation. EDS point analyses were carried out on representative microstructural regions, and the measured elemental proportions were compared with expected phase stoichiometries and relevant Al–Mg and Mg–Si phase diagrams, together with SEM morphology, to infer phase assignments in Table 2 and Table 3.

To further analyze the effects of Sc addition on the major interfacial phases and crystallographic characteristics, representative specimens were examined by electron backscatter diffraction (EBSD). The EBSD specimens were mechanically polished and then subjected to low-energy ion-beam surface treatment to reduce surface damage introduced during mechanical preparation. Data were acquired using an Oxford Symmetry system and processed with Channel 5 software to obtain interfacial phase maps, inverse pole figure (IPF) maps, and kernel average misorientation (KAM) maps. Based on the phase maps, the sizes of the interfacial microstructural units of the indexed phases and the Mg_2_Si particles were quantified as equivalent circular diameters directly using AZtecCrystal software (version 2.1, build 2.1.259; Oxford Instruments, Buckinghamshire, UK); the mean values, maximum values, and standard deviations were then compared. In this study, the features included in the equivalent circular diameter statistics derived from the phase maps are collectively referred to as “interfacial microstructural units of the indexed phases.” The EBSD scan step size used for the reported phase maps was 3.5 μm. Size measurements for small features may be affected by spatial sampling and phase-boundary segmentation. The minimum grain-size setting for the reported size statistics was 10 pixels. Standard deviations represent the dispersion of the measured feature sizes, not the instrumental measurement error. All reported interface-thickness and equivalent-circular-diameter statistics in the main text (including means, maxima, and standard deviations) are rounded to the nearest micrometer. Percentage changes in the size statistics are calculated from the displayed integer values to ensure consistency with the size-distribution results presented later; the original measurements and EBSD datasets are unchanged.The EBSD scan step size of 3.5 μm is an acquisition parameter and is not rounded.

### 2.3. Vickers Hardness Testing

The Vickers hardness distribution along the interface normal of the Al/Mg bimetallic composites with different nominal Sc additions was measured using a 200HV-5 hardness tester (Wuhan Huayin Testing Instrument Co., Ltd., Wuhan, China). One specimen from each group was examined, with three separate indentation profiles measured at different circumferential positions on the annular interface of each specimen. The testing path extended from the A356 side across the interfacial region to the AZ91D side, with a spacing of 150 μm, a test load of 2.94 N (HV0.3), and a dwell time of 15 s. The three indentation profiles were used to obtain the reported mean hardness distribution and standard-deviation error bars (*n* = 3 paths); the measurements represent within-specimen spatial replication rather than independent specimen replication. When an indentation crossed two different microstructural regions, the measurement point was assigned to the region occupying the larger portion of the indentation to reduce ambiguity associated with the interfacial boundary. The indentation loading speed was not separately recorded.

## 3. Results

### 3.1. Microstructure of the Al/Mg Bimetallic Composites

**Figure 2:**
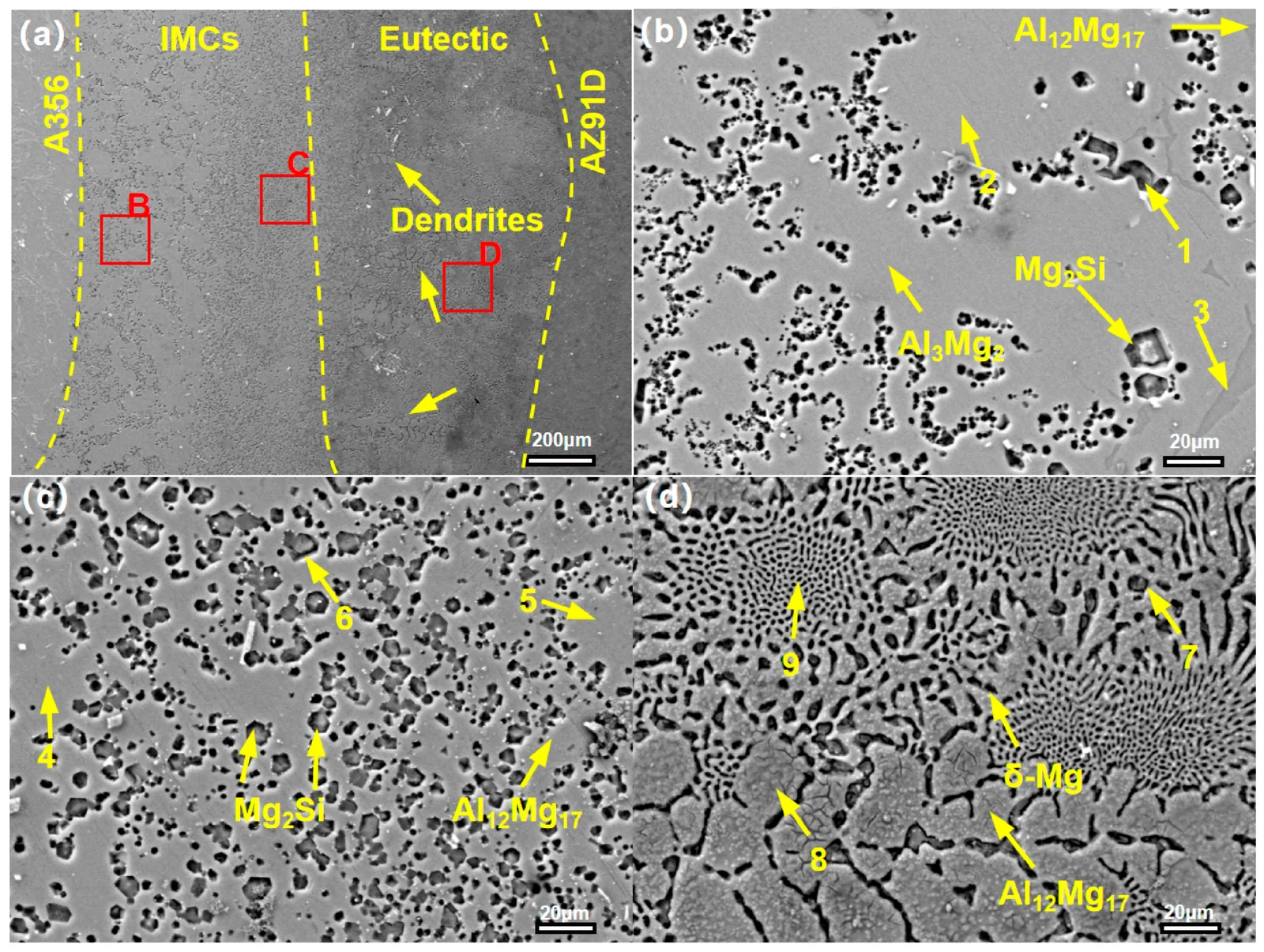
Interfacial microstructure and EDS point-analysis locations of the Al/Mg bimetallic composite without Sc (Group A): (**a**) overall interface morphology; (**b**) Al_3_Mg_2_ interlayer; (**c**) Al_12_Mg_17_-dominated IMC region; and (**d**) eutectic region. Numbered markers identify the EDS point-analysis locations; the corresponding measured compositions and inferred phase assignments are listed in Table 2.The red boxes labeled B, C, and D in (**a**) indicate the regions enlarged in (**b**), (**c**), and (**d**), respectively.

Figure 2a shows the low-magnification morphology of the Al/Mg bimetallic interface in Sc-free Group A. A continuous transition region formed between Al and Mg, with distinct microstructural partitioning across the interface. Figure 2b–d show the corresponding high-magnification SEM morphologies, and the EDS point-analysis results for the marked locations are listed in Table 2. Based on the EDS results and phase-equilibrium data, the major interfacial microstructures were preliminarily identified. Al–Mg reaction phases were identified primarily with reference to thermodynamic assessments and phase-diagram calculations of the Al–Mg system [10,11,12], whereas Mg_2_Si was identified with reference to the Al–Mg–Si phase-equilibrium relationship [13] and supplementary ASM phase-diagram data [14,15]. According to the morphological and compositional differences, the interface was divided into an intermetallic compound (IMC) layer and a eutectic layer. The IMC layer was further divided into a β region dominated by the Al_3_Mg_2_ interlayer and a γ region dominated by Al_12_Mg_17_. The region in Figure 2b mainly consisted of an Al_3_Mg_2_ matrix and Mg_2_Si particles; the region in Figure 2c mainly consisted of an Al_12_Mg_17_ matrix containing a relatively large number of Mg_2_Si particles; and the eutectic region in Figure 2d mainly consisted of a δ-Mg solid solution, a network-like eutectic microstructure, and secondary γ-Al_12_Mg_17_ dendrites. This interfacial partitioning is generally consistent with observations reported for Al-xSi/AZ91D compound-cast composites [8]. These results provide a baseline for subsequent comparison of the interfacial changes induced by different Sc additions.

**Figure 3:**
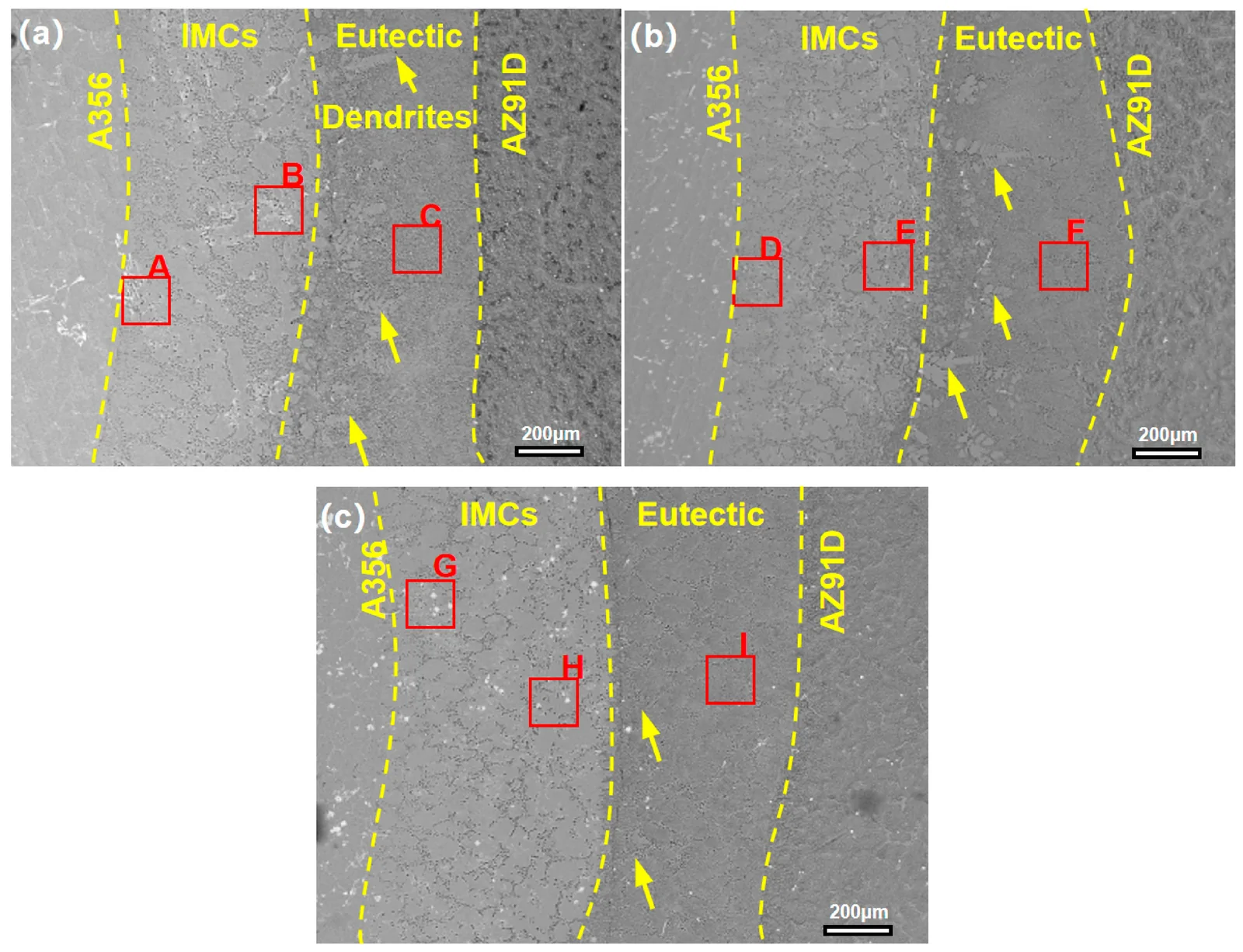
Low-magnification SEM morphologies of the Al/Mg bimetallic interfaces with different nominal Sc additions: (**a**) 0.6 wt.% Sc (S1); (**b**) 0.9 wt.% Sc (S2); and (**c**) 1.2 wt.% Sc (S3).The regions labeled A–I in Figure 3 correspond to the enlarged micrographs shown in Figure 4a–i, respectively.

Figure 3 shows the low-magnification SEM morphologies of the Al/Mg bimetallic interfaces in Groups S1, S2, and S3. All three specimens retained the interfacial partitioning into an IMC layer and a eutectic layer; however, the total interface thickness and the morphologies of the locally bright Sc-enriched regions varied with nominal Sc addition. According to the EDS point analyses, some bright regions mainly contained Al and Sc, whereas others also contained appreciable Mg. Considering the EDS electron-interaction volume and the influence of adjacent microstructures, not all Sc-enriched regions were identified as Al_3_Sc in this study; instead, they are collectively described as “Sc-enriched regions.” Values were rounded individually to the nearest micrometer for reporting; consequently, the sum of the displayed rounded layer thicknesses may differ from the separately rounded total by 1 μm. Thickness measurements showed that the average thicknesses of the IMC and eutectic layers in Group A were 981 μm and 770 μm, respectively, giving a total interface thickness of 1750 μm. After Sc addition, the corresponding layer thicknesses in Group S1 were 562 μm and 507 μm, yielding a total thickness of 1069 μm, which represented a reduction of approximately 39% relative to Group A. In Group S2, the two layer thicknesses were 523 μm and 565 μm, respectively, giving a total thickness of 1088 μm, a reduction of approximately 38%. In Group S3, the corresponding values were 497 μm and 425 μm, giving a total thickness of 922 μm, a reduction of approximately 47%. Thus, the IMC-layer thickness, eutectic-layer thickness, and total interface thickness were lower in all three Sc-containing specimens than in Group A, indicating a clear association between Sc microalloying on the Al side and interfacial thinning. Notably, the total interface thickness did not decrease strictly monotonically with increasing Sc content: S2 was slightly thicker than S1, whereas S3 exhibited the thinnest interface. This result indicates that the total interface thickness was influenced not only by Sc content but also by the local microstructure and reaction state.

**Figure 4:**
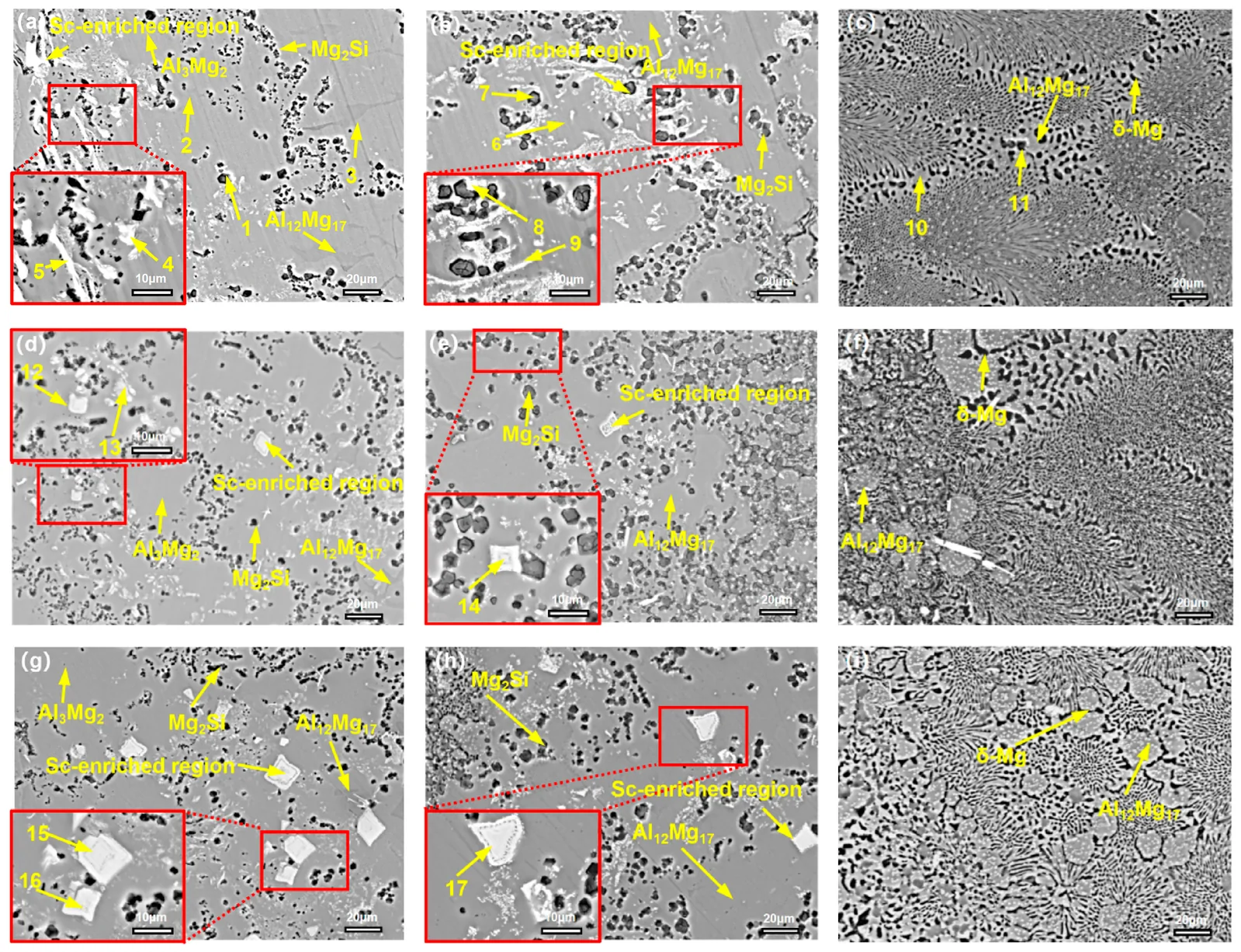
High-magnification SEM microstructures and EDS point-analysis locations of the Al/Mg interfaces with different Sc additions: (**a**–**c**) 0.6 wt.% Sc (S1); (**d**–**f**) 0.9 wt.% Sc (S2); and (**g**–**i**) 1.2 wt.% Sc (S3). Numbered markers indicate the EDS point-analysis locations; the corresponding compositions and inferred phase/region assignments are listed in Table 3.

Figure 4 shows the high-magnification SEM morphologies of the Al/Mg interfaces in Groups S1, S2, and S3, and the EDS point-analysis results for the marked locations are listed in Table 3. The interfacial microstructure of Group S1 is shown in Figure 4a–c. The Mg_2_Si particles in the IMC region were markedly smaller than those in the Sc-free specimen and were distributed more uniformly, with a pronounced reduction in locally coarse particles. Group S1 therefore exhibited clear Mg_2_Si refinement relative to the Sc-free specimen. In the eutectic region, both the number and size of coarse *γ*-Al_12_Mg_17_ dendrites decreased markedly, and some dendrites exhibited fine short-rod-like or locally discontinuous morphologies, resulting in a finer and more uniform overall eutectic microstructure. In addition, numerous Sc-enriched regions were observed near the interface. These regions were mainly dispersed with filamentous or flocculent morphologies, without obvious large-scale local agglomeration.

The interfacial microstructure of Group S2 is shown in Figure 4d–f. Mg_2_Si in the IMC region remained relatively dispersed; however, its refinement was less pronounced than in Group S1, and relatively large Mg_2_Si particles were still observed in some areas. In the eutectic region, the number of coarse *γ*-Al_12_Mg_17_ dendrites decreased further, and some dendrites exhibited short-rod-like or fragmented morphologies with reduced continuity. Meanwhile, the morphology of the Sc-enriched regions near the interface changed. In addition to filamentous or flocculent Sc-enriched regions, blocky Sc-enriched regions appeared locally, resulting in the coexistence of filamentous/flocculent and blocky morphologies. These observations indicate that, with increasing nominal Sc addition, the Sc-enrichment morphology began to evolve from a relatively dispersed state toward localized blocky enrichment.

The interfacial morphology of Group S3 is shown in Figure 4g–i. Compared with Group S1, Mg_2_Si refinement was less pronounced, and the particle distribution was relatively sparse in some areas. Coarse *γ*-Al_12_Mg_17_ dendrites remained limited in the eutectic region, where finer or locally discontinuous microstructures were observed. At the same time, the number of blocky Sc-enriched regions increased, and local enrichment became more pronounced. Considering all three Sc-addition levels, the morphology of the Sc-enriched regions near the interface generally evolved from relatively dispersed filamentous/flocculent features to the coexistence of filamentous/flocculent and blocky features, accompanied by increased blocky enrichment. It should be emphasized that EDS point analysis provides only local elemental-composition information and cannot independently determine crystal structure. Points 4, 5, and 14 in Table 3 mainly contained Al and Sc, and their mass fractions were close to the theoretical composition of Al_3_Sc (approximately 64.3 wt.% Al and 35.7 wt.% Sc); therefore, they are described here as Al_3_Sc-related phases. The other points containing appreciable Mg are described as Mg-containing Sc-enriched regions. Because the EDS interaction volume may simultaneously include an Sc-enriched particle and adjacent Mg-rich microstructures, the presence of an Mg signal does not directly exclude the possible presence of an Al_3_Sc-related phase. Confirmation of the crystal structure would require further characterization by techniques such as transmission electron microscopy/selected-area electron diffraction (TEM/SAED).

### 3.2. Effect of Sc on the Interfacial Microstructural Characteristics of Al/Mg Bimetallic Composites

To further analyze the effect of Sc on the interfacial microstructure, Groups A and S1 were selected for EBSD, elemental mapping, and microstructural size statistics. Group S1 was selected primarily because, in addition to exhibiting a marked reduction in total interface thickness, it showed the clearest Mg_2_Si refinement and a more dispersed Sc-enrichment morphology among the three Sc-addition levels, thereby facilitating comparison of the major phases, crystallographic state, elemental distribution, and microstructural scale before and after Sc introduction. It should be noted that Group S3 exhibited the minimum total interface thickness. Therefore, the detailed comparison between Groups S1 and A was intended mainly to evaluate microstructural refinement and changes in elemental distribution and does not represent the complete evolution across all nominal Sc additions.

#### 3.2.1. Interfacial Phase Constitution and Crystallographic Characteristics

**Figure 5:**
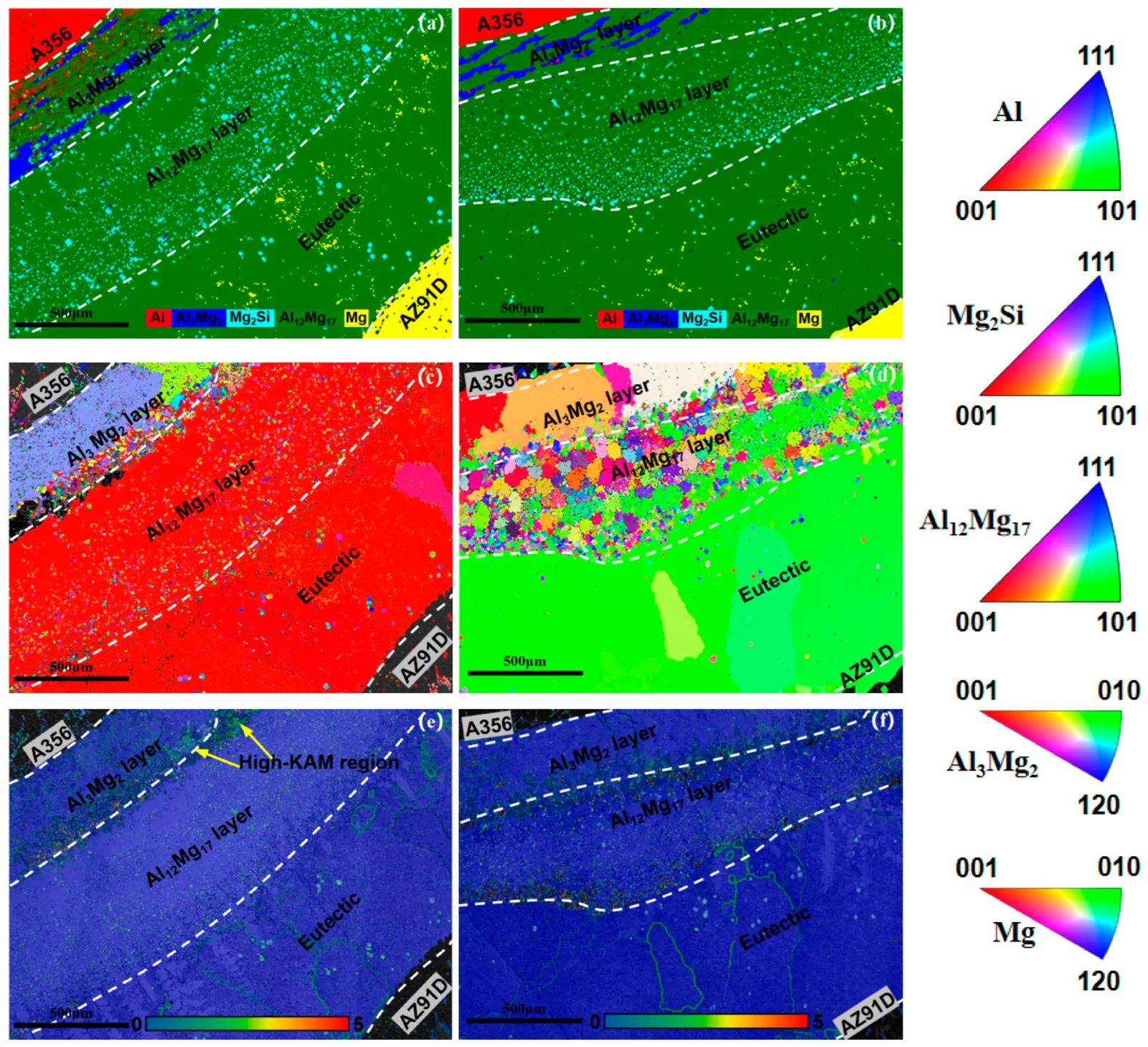
EBSD results for the Al/Mg interfaces of the Sc-free and 0.6 wt.% Sc specimens: (**a**,**b**) phase maps; (**c**,**d**) inverse pole figure (IPF) maps; and (**e**,**f**) kernel average misorientation (KAM) maps. Panels (**a**,**c**,**e**) correspond to the Sc-free specimen, and panels (**b**,**d**,**f**) correspond to the 0.6 wt.% Sc specimen.

Figure 5 presents the EBSD results for the Al/Mg interfaces of the Sc-free and 0.6 wt.% Sc specimens. Figure 5a,b show the phase maps of the two specimens, Figure 5c,d show the corresponding inverse pole figure (IPF) maps, and Figure 5e,f show the kernel average misorientation (KAM) maps. The analyzed regions covered the Al matrix, IMC layer, eutectic layer, and Mg matrix, thereby providing a relatively complete representation of the interfacial phase constitution and crystallographic characteristics before and after Sc addition.

As shown in the phase maps in Figure 5a,b, both specimens exhibited a distinct layered interfacial structure consisting, from the Al side to the Mg side, of the Al matrix, IMC layer, eutectic layer, and Mg matrix. The major phases that could be reliably indexed by EBSD included Al, Mg, Al_3_Mg_2_, Al_12_Mg_17_, and Mg_2_Si, consistent overall with the principal interfacial microstructural features identified by SEM-EDS. After the addition of 0.6 wt.% Sc, no marked change was observed in the types of major reaction phases that could be reliably indexed. The Sc-enriched regions observed by SEM-EDS were also not consistently identified in the conventional EBSD phase maps. Therefore, the statement that “the major phase types did not change markedly” refers only to the principal phases that could be reliably indexed under the present EBSD conditions. Compared with Group A, the phase map of Group S1 contained fewer large microstructural units, and the interfacial microstructure exhibited an overall refinement trend. This finding indicates that the effect of Sc was manifested primarily as changes in the size and morphology of the existing interfacial microstructures rather than as the formation of abundant new reaction phases that could be reliably indexed by EBSD.

No Al_3_Sc phase could be reliably indexed in the EBSD phase map of Group S1. EBSD phase identification is affected by factors such as spatial resolution, pattern quality, and the size and volume fraction of second phases, as discussed in the relevant EBSD methodology [58]. Spatial resolution and indexing quality are also key limitations in the reliable characterization of fine grains and substructures [59]. Considering that the volume fraction of Sc-related second phases in the analyzed region may have been low and that some precipitates may have been smaller than the reliable indexing scale of conventional EBSD, the presence of Al_3_Sc cannot be established solely from the present EBSD results. Accordingly, the failure to index Al_3_Sc by EBSD is not regarded as evidence of its absence, and the EBSD data were not used to quantify its content. The discussion of Al_3_Sc-related phases is therefore limited to cautious inference based on the EDS compositions and literature reports.

Figure 5c,d show the inverse pole figure (IPF) maps of the two specimens. Multiple orientation colors were present in the different interfacial regions, and no distinct single preferred orientation was observed. After the addition of 0.6 wt.% Sc, no significant change was observed in the overall orientation distribution. Thus, the IPF results indicate that Sc addition did not induce an identifiable change in the overall preferred orientation, whereas changes in microstructural scale were quantitatively evaluated using the phase maps and subsequent size statistics.

Figure 5e,f show the kernel average misorientation (KAM) distributions of Groups A and S1. KAM represents the local misorientation between neighboring measurement points and can be used to describe local orientation gradients; its measurement accuracy and the definition of local gradients have been systematically discussed [60]. EBSD-based strain-analysis studies have shown that local misorientation can reflect features associated with lattice curvature and plastic deformation but cannot be directly equated with macroscopic stress [61]. The relationship between orientation gradients and geometrically necessary dislocations has also been investigated using two-dimensional and three-dimensional EBSD [62]. Both specimens were dominated by low-KAM regions, whereas relatively high KAM values were mainly distributed near certain grain boundaries, phase boundaries, and interfacial reaction regions. The relationship between local misorientation near grain boundaries and damage/deformation characterization has been reported elsewhere [63]. The average KAM value was approximately 0.24° for Group A and 0.27° for Group S1, indicating only a slight increase. In conjunction with the phase-map results, the increased number of phase and microstructural boundaries associated with refinement in Group S1 may have contributed to a modest adjustment of the local orientation gradients. Because error ranges and significance tests were unavailable, the difference between 0.24° and 0.27° should not be interpreted as statistically significant. In this study, the KAM results are used only as supplementary evidence of a slight adjustment in the local crystallographic state after Sc addition.

Taken together, the phase-map, IPF, and KAM results show that, after the addition of 0.6 wt.% Sc, neither the major interfacial phases that could be reliably indexed nor the overall preferred orientation changed markedly, while only a slight adjustment in the local misorientation distribution was observed. The more evident change was the reduction in coarse microstructural units. This microstructural change is further examined below using elemental mapping and size statistics.

#### 3.2.2. Interfacial Elemental Distribution

**Figure 6:**
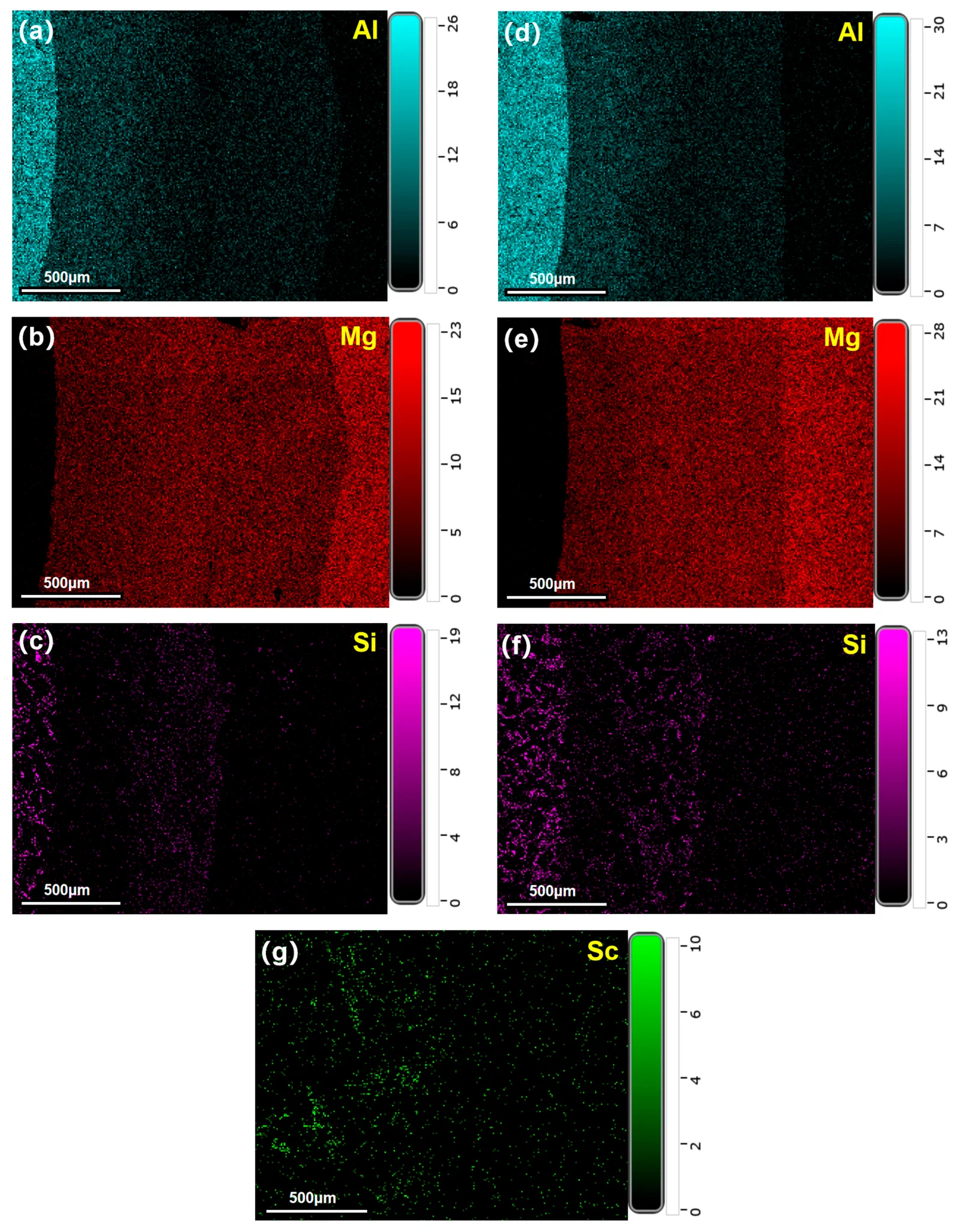
Elemental maps of the Al/Mg interfaces of the Sc-free and 0.6 wt.% Sc specimens: (**a**–**c**) Al, Mg, and Si distributions in the Sc-free specimen; and (**d**–**g**) Al, Mg, Si, and Sc distributions in the 0.6 wt.% Sc specimen.

To further analyze changes in the spatial elemental distribution at the interface before and after Sc addition, elemental mapping was performed for Groups A and S1, as shown in Figure 6. Figure 6a–c show the Al, Mg, and Si distributions in Group A, respectively, whereas Figure 6d–g show the Al, Mg, Si, and Sc distributions in Group S1, respectively.

As shown in Figure 6a,b, Al and Mg exhibited distinct complementary distributions on the two sides of the interface in Group A. The Al signal was stronger on the A356 side and gradually decreased toward the interior of the interface, whereas Mg was mainly distributed on the AZ91D side and decreased markedly near the Al matrix. A compositional transition zone of finite width was present near the interface, indicating substantial Al/Mg elemental migration during compound casting. This behavior is consistent with the interfacial interdiffusion characteristics reported for Al-xSi/AZ91D compound-cast composites [8]. This transition zone was also the principal region in which Al–Mg reaction phases such as Al_3_Mg_2_ and Al_12_Mg_17_ formed, and the stability of these phases can be explained using Al–Mg thermodynamic and phase-diagram data [10,11,12]. Figure 6c shows that Si was mainly concentrated on the Al side and in the adjacent interfacial region and extended into the interface as discontinuous point-like features and local enrichments. Combined with the SEM observations and phase analysis, these Si-enriched regions corresponded qualitatively to the distribution of Mg_2_Si particles. The Al–Mg–Si phase-equilibrium relationship supports the formation of Mg_2_Si from Si and Mg under appropriate local compositional conditions [13].

**Figure 7:**
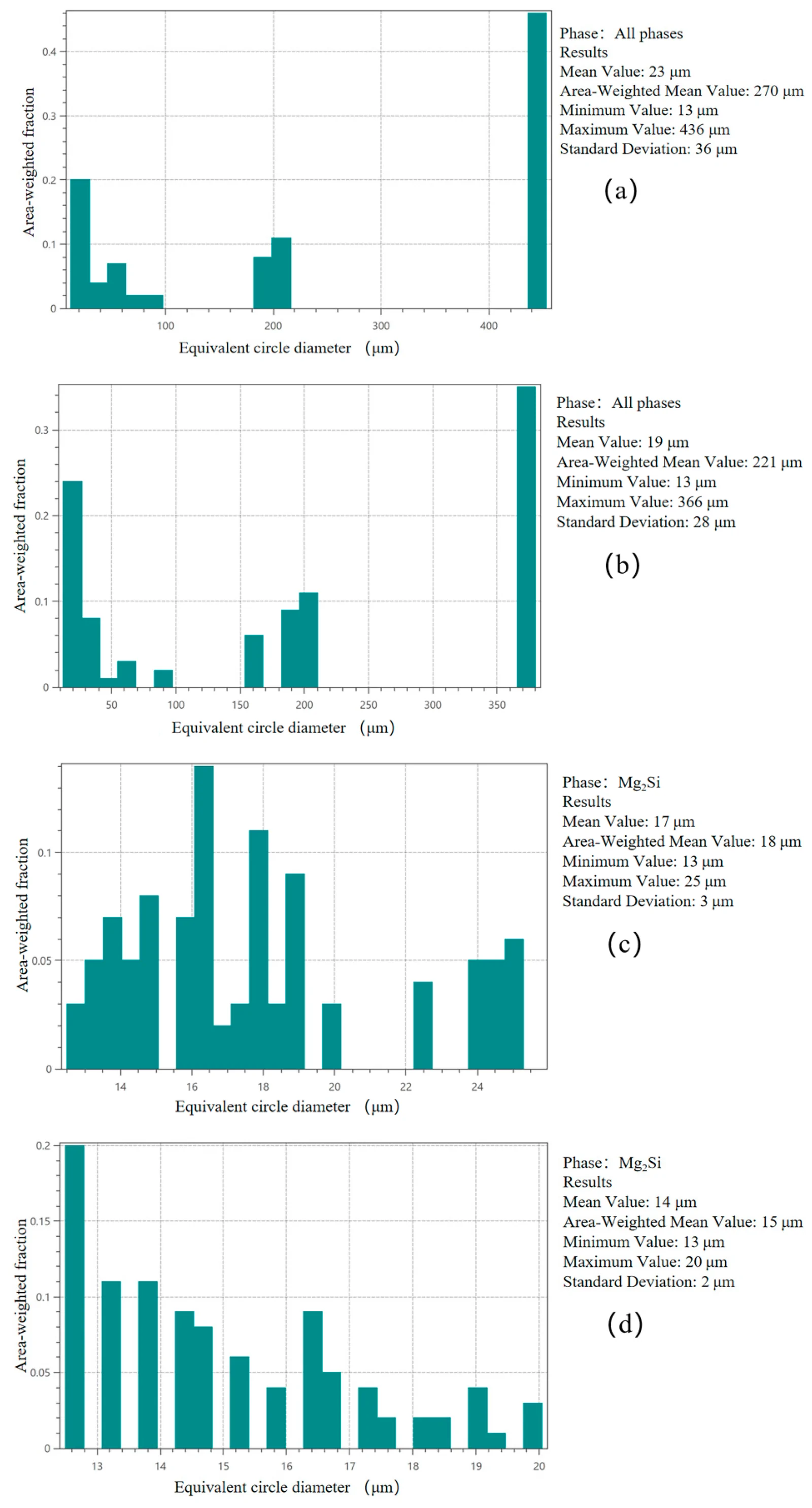
Size statistics of the interfacial microstructure in the Sc-free and 0.6 wt.% Sc specimens: (**a**) equivalent circular diameter distribution of all indexed phases in the Sc-free specimen; (**b**) equivalent circular diameter distribution of all indexed phases in the 0.6 wt.% Sc specimen; (**c**) equivalent circular diameter distribution of Mg_2_Si particles in the Sc-free specimen; and (**d**) equivalent circular diameter distribution of Mg_2_Si particles in the 0.6 wt.% Sc specimen. Reported standard deviations describe feature-size dispersion, not measurement uncertainty. The means, maxima, and standard deviations discussed in the text are reported as integers consistent with the displayed Figure 7 statistics; percentage changes are calculated from these displayed rounded values. The area-weighted mean diameters shown in panels (**a**–**d**) are 270, 221, 18, and 15 μm, respectively; these are distinct from the arithmetic mean diameters used for the percentage comparisons in the text.

After the addition of 0.6 wt.% Sc, the overall Al and Mg distribution pattern remained unchanged (Figure 6d,e): Al was concentrated on the A356 side, Mg on the AZ91D side, and a compositional transition region remained between them. Compared with Group A, this transition region appeared more confined in Group S1, consistent with the reduced interface thickness measured by SEM. Because concentration gradients and diffusion coefficients were not quantified, the mapping results demonstrate a narrower interfacial reaction range, not a directly measured decrease in atomic diffusivity. In Figure 6f, the Si-enriched features within the interface were finer and more dispersed. This observation agrees with the Mg_2_Si statistics in Figure 7, for which the mean equivalent circular diameter decreased from 17 to 14 μm and the maximum decreased from 25 to 20 μm. Figure 6g further shows that Sc occurred as discrete enrichments rather than as a continuous interfacial layer. Thus, the observed thinning is inconsistent with a simple continuous-barrier mechanism and instead points to an indirect effect of Sc on the Al-side microstructural state, local solute distribution, and subsequent reaction/solidification conditions.

Overall, the elemental maps show that Sc addition did not change the basic Al/Mg compositional arrangement. The key changes in Group S1 were a narrower reaction range, finer and more dispersed Si-enriched features, and discrete Sc enrichment. Together with the SEM and size-statistics results, these observations support interfacial confinement and Mg_2_Si refinement while avoiding an unsupported claim that Sc directly lowers the Al/Mg diffusion coefficient.

#### 3.2.3. Size Statistics of the Interfacial Microstructure and Mg_2_Si Particles

To quantitatively compare the interfacial microstructural scale before and after Sc addition, the equivalent circular diameters of the interfacial microstructural units of the indexed phases and the Mg_2_Si particles in Groups A and S1 were analyzed, as shown in Figure 7. Figure 7a,b show the equivalent circular diameter distributions of the interfacial microstructural units in the two specimens, whereas Figure 7c,d show the corresponding distributions for Mg_2_Si particles. The average equivalent circular diameter of the interfacial microstructural units was 23 μm in Group A and decreased to 19 μm in Group S1, corresponding to a reduction of approximately 17%. The maximum equivalent circular diameter decreased from 436 μm to 366 μm, corresponding to a reduction of approximately 16%. These results indicate that the addition of 0.6 wt.% Sc reduced the overall scale of the interfacial microstructure and, in particular, reduced the occurrence of large microstructural units.

In addition to the mean and maximum values, the dispersion of the interfacial microstructural sizes also changed. The standard deviation of the equivalent circular diameter of the interfacial microstructural units decreased from 36 μm in Group A to 28 μm in Group S1. The lower standard deviation indicates that the size difference between large and small microstructural units decreased and that the size distribution became more concentrated. Together with the simultaneous decreases in the mean and maximum values, these results indicate that 0.6 wt.% Sc not only reduced the average scale of the interfacial microstructure but also decreased the occurrence of coarse microstructural units, thereby producing a more uniform size distribution.

Based on the overall microstructural statistics, the Mg_2_Si particles were analyzed separately. The average equivalent circular diameter of Mg_2_Si decreased from 17 μm in Group A to 14 μm in Group S1, corresponding to a reduction of approximately 18%, while the maximum equivalent circular diameter decreased from 25 μm to 20 μm, corresponding to a reduction of approximately 20%. The standard deviation simultaneously decreased from 3 μm to 2 μm, corresponding to a reduction of approximately 33%. The decreases in the mean, maximum, and standard deviation indicate that the Mg_2_Si particles were refined after the addition of 0.6 wt.% Sc, with a lower proportion of coarse particles and a more concentrated size distribution.

As shown collectively in Figure 7a–d, the microstructural refinement associated with 0.6 wt.% Sc was not limited to a single phase. The average equivalent circular diameter of the interfacial microstructural units decreased from 23 μm to 19 μm, whereas that of Mg_2_Si decreased from 17 μm to 14 μm; the maximum sizes and standard deviations of both statistical populations also decreased. These quantitative results support the reduction in coarse microstructural features observed by SEM and EBSD.

Accordingly, the comparison between Groups A and S1 shows that 0.6 wt.% Sc primarily changed the scale and morphology of existing interfacial constituents rather than the major phase constitution. The reduction in coarse indexed microstructural units, the refinement of Mg_2_Si, and the more confined elemental transition range collectively indicate that Sc regulates phase growth and local solidification within the existing Al/Mg reaction framework.

### 3.3. Interfacial Hardness Distribution

**Figure 8:**
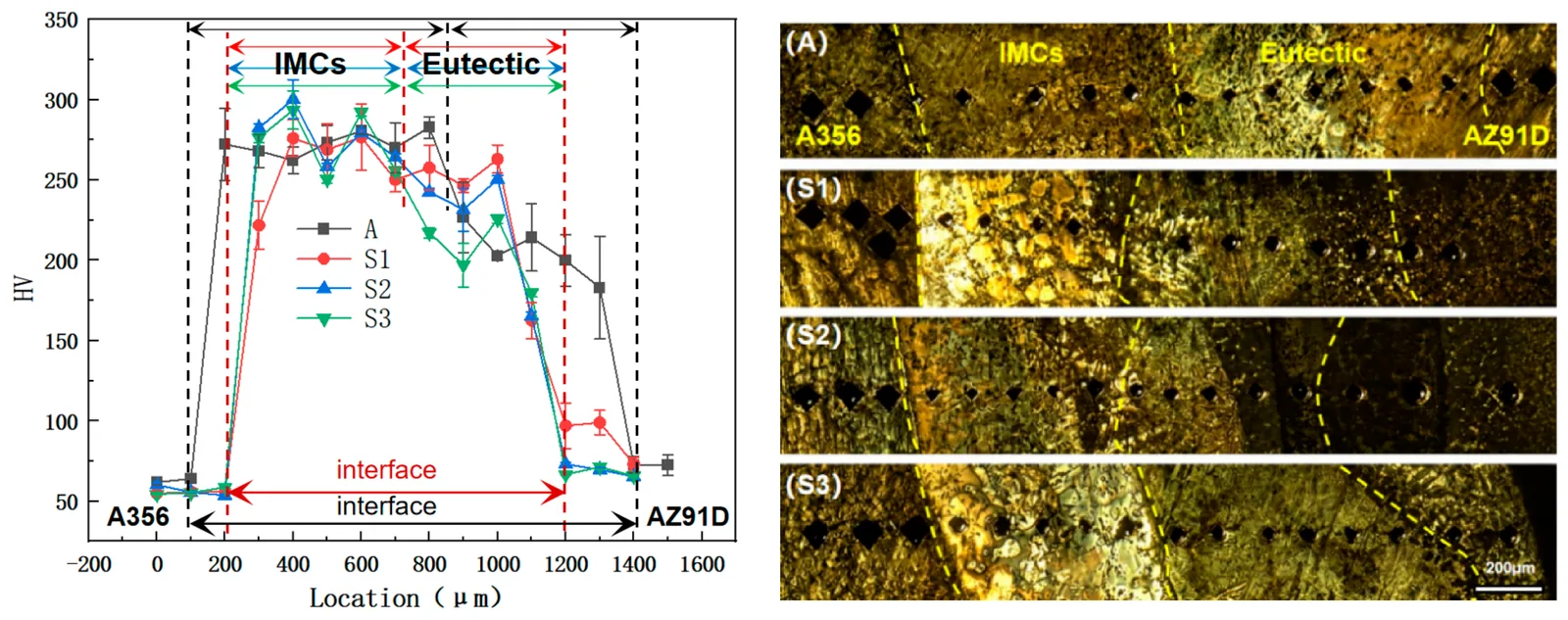
Vickers hardness distribution and indentation morphologies across the Al/Mg bimetallic interfaces with different Sc additions: (**left**) hardness distribution from the A356 side to the AZ91D side; and (**right**) interfacial micro-indentation morphologies of Groups A, S1, S2, and S3.The four subfigures (A-S3) correspond to samples A, S1, S2, and S3, respectively. In each subfigure, the regions from left to right are the A356 aluminum alloy, intermetallic compound (IMC) layer, eutectic layer, and AZ91D magnesium alloy.

To characterize the local hardness response of the Al/Mg interface at different nominal Sc additions, Vickers hardness tests were performed on Groups A, S1, S2, and S3, as shown in Figure 8. The testing path extended from the A356 side across the interfacial region to the AZ91D side. All specimens exhibited a pronounced hardness gradient: the Al and Mg matrix regions showed relatively low hardness, whereas the interfacial region exhibited markedly higher values. Indentation studies of dissimilar Al/Mg interfaces have shown that reaction layers can exhibit local hardness substantially higher than that of the two substrates [16]. Studies of compound casting and solid–liquid cast rolling have also shown that interfacial regions enriched in Al–Mg intermetallic compounds are generally associated with higher hardness and lower plasticity/toughness [8,9]. In Group A, the hardness was approximately 60 HV on the Al side, increased rapidly upon entering the interface, and remained relatively high over approximately 200–800 μm, reaching a peak value of approximately 280 HV. This behavior indicates the presence of a relatively wide high-hardness interfacial region in the Sc-free specimen.

After Sc addition, the spatial extent of the high-hardness region in Groups S1, S2, and S3 was reduced relative to Group A, consistent with the microstructural results showing decreases in IMC-layer thickness and total interface thickness. However, Sc addition did not produce a corresponding overall decrease in interfacial hardness; the interfaces of all Sc-containing specimens still maintained relatively high hardness values of approximately 250–300 HV. Thus, the primary effect of Sc was reflected in the width and spatial distribution of the high-hardness interfacial region rather than in a simple change in the intrinsic hardness of the hard phases. The peak hardness did not vary monotonically with nominal Sc addition: Group S2 retained relatively high hardness, whereas some regions in Group S3 exhibited lower values, indicating that local phase constitution and microstructural scale also affected the measured hardness. In the indentation morphologies, the indentations within the interfacial region were visibly smaller than those in the Al and Mg matrix regions, consistent with the higher interfacial hardness.

Overall, the hardness results showed a clear correspondence with the changes in interface thickness. After Sc addition, the high-hardness interfacial region narrowed as the interface became thinner, while locally high hardness was retained within the interface. Therefore, the hardness results primarily reflect Sc-induced changes in the spatial distribution of the interfacial microstructure and the associated local mechanical response and should not be directly equated with an improvement in interfacial bonding strength or overall mechanical properties.

## 4. Discussion

### 4.1. Formation Mechanism of the Al/Mg Bimetallic Interfacial Microstructure

During lost foam compound casting, the high-temperature AZ91D melt comes into direct contact with the A356 insert, and rapid heat transfer and local reactions initially occur at the interface. This overall process is consistent with the established understanding of Mg/Al compound casting [1,8]. Subsequently, Al and Mg migrate within the interfacial region, and reaction phases such as Al_3_Mg_2_ and Al_12_Mg_17_ form when the local composition satisfies the relevant conditions. The stability of these phases can be explained by Al–Mg thermodynamic data [10,11,12], while the possible pathway by which Si participates in Mg_2_Si formation is supported by the Al–Mg–Si phase-equilibrium relationship [13]. As the temperature continues to decrease, the interfacial reaction region and the remaining liquid enter the solidification and phase-transformation stages, which can be interpreted using general theories of phase transformations in metals and alloys [64] and solidification [65], respectively. Combined with the SEM/EDS results of the present study, formation of the Sc-free interface can be summarized in three successive stages: first, the surface layer of the Al insert is heated and a local reaction region forms; second, Al, Mg, Si, and other elements migrate within this region, leading to the formation of Al–Mg intermetallic compounds and Mg_2_Si; finally, as the temperature decreases, the Mg side and the residual liquid at the interface solidify to form the eutectic microstructure.

**Figure 9:**
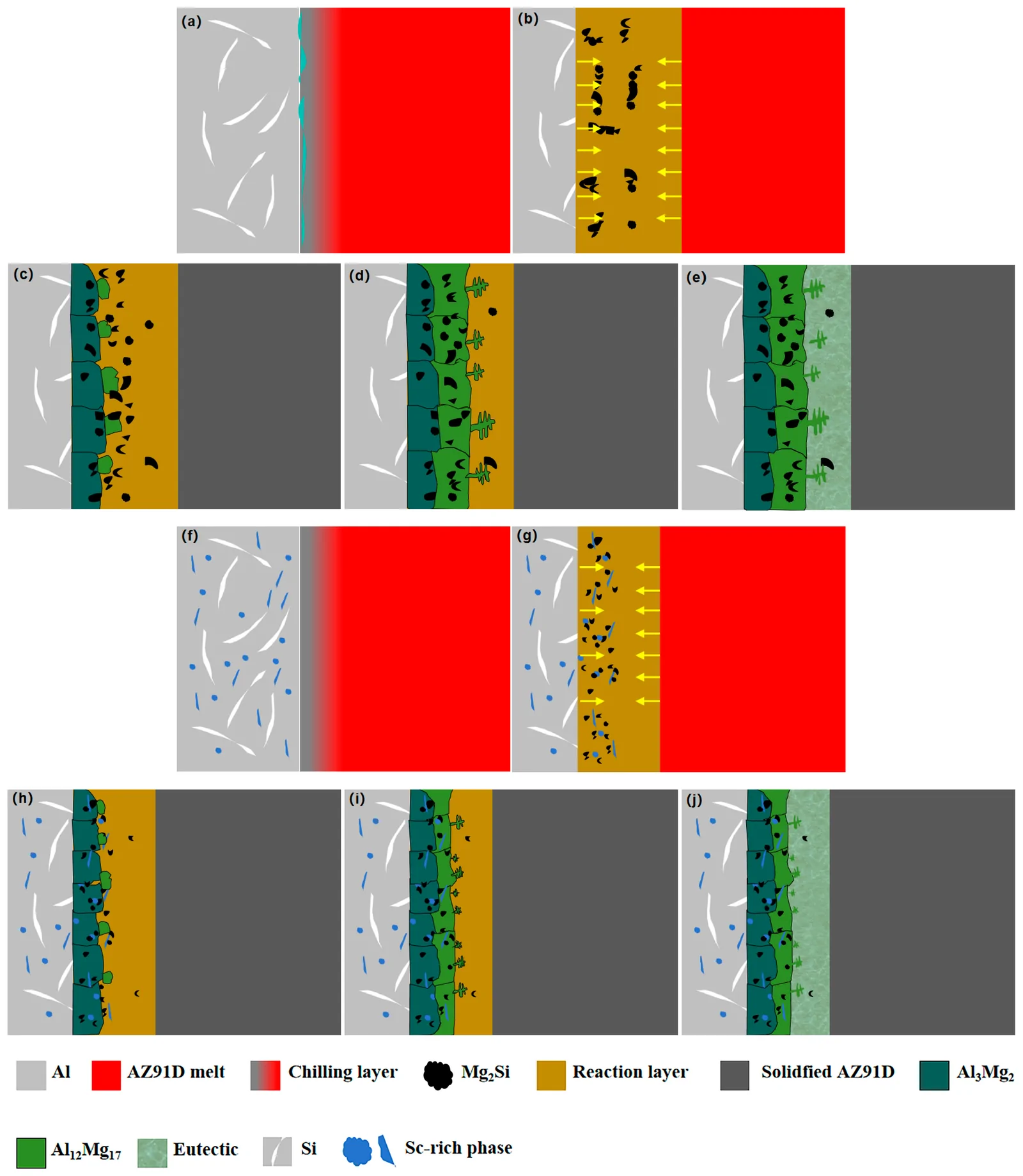
Schematic illustration of Al/Mg bimetallic interface formation and Sc-mediated regulation: (**a**–**e**) formation process of the Sc-free interface; and (**f**–**j**) formation process after Sc microalloying of the Al insert. In the schematic, blue features denote Sc-enriched regions rather than a crystallographically confirmed Sc-containing phase.

As illustrated in Figure 9a,b, during the initial stage of pouring, the high-temperature Mg melt comes into contact with the lower-temperature Al insert. Heat is transferred from the Mg melt to the Al side, causing the surface layer of the insert to heat rapidly and undergo local melting, thereby forming the initial interfacial reaction region. Al and Mg then migrate within this region, while Si from the Al side also enters the interface; the Al–Mg–Si phase-equilibrium data support the possibility of Mg_2_Si formation from Si and Mg [13]. As the local composition continues to evolve, conditions favorable for the formation of phases such as Al_3_Mg_2_ and Al_12_Mg_17_ are progressively established. This interpretation is supported by thermodynamic calculations of the Al–Mg system [10,11,12], with ASM phase-diagram data providing supplementary criteria [14,15], as illustrated in Figure 9c,d. The sequence shown in Figure 9 summarizes the main microstructural evolution pathway and does not imply that Mg_2_Si and Al–Mg intermetallic compounds form in completely separate, strictly sequential steps during rapid solidification. With further cooling, the Mg side and the residual liquid gradually solidify, forming a eutectic microstructure dominated by Mg solid solution and *γ*-Al_12_Mg_17_ between the IMC layer and the Mg matrix. Similar microstructures have been reported in Al/Mg compound-casting studies [8,26,27], and their solidification evolution can be interpreted using general phase-transformation and solidification theories [64,65]. A continuous graded interface, as shown in Figure 9e, is ultimately obtained.

The Sc-free interface can therefore be summarized as follows: melt/insert contact and rapid heat transfer → local melting of the Al surface and multielement migration → formation and growth of Al–Mg intermetallic compounds and Mg_2_Si → solidification of the remaining interfacial liquid into the eutectic region. This sequence provides the reference state for identifying where Al-side Sc microalloying acts.

### 4.2. Mechanism of Al-Side Sc Microalloying in Interfacial Regulation

Sc did not replace the basic Al/Mg interface-formation pathway. Instead, the experimental evidence identifies three coupled effects: (i) all Sc-containing specimens had thinner IMC and eutectic layers; (ii) coarse γ-Al_12_Mg_17_ dendrites were suppressed; and (iii) Mg_2_Si refinement was strongest at 0.6 wt.% Sc. At the same time, EBSD showed no marked change in the major reliably indexed phases, and elemental mapping showed no continuous Sc-rich layer. Sc therefore acts mainly by regulating the extent and morphology of the existing reaction/solidification process rather than by creating a new dominant reaction pathway.

Because Sc was introduced into the A356 insert before compound casting, its earliest influence is on the Al side. Upon contact with the AZ91D melt, local heating and partial melting of the Al surface still occur, followed by Al/Mg/Si redistribution and formation of Al_3_Mg_2_, Al_12_Mg_17_, and Mg_2_Si (Figure 9f–h). However, all three Sc-containing groups exhibited a smaller reaction-zone thickness, and Group S1 showed a more confined Al/Mg transition range. These observations are consistent with Sc-induced changes in the Al-side microstructural/chemical state that limit continued expansion of the reaction region; they do not require, and the mapping results do not support, a continuous Sc-rich diffusion barrier.

During subsequent cooling (Figure 9i,j), the effect of Sc becomes evident in the solidification products. The amount of coarse γ-Al_12_Mg_17_ dendritic microstructure decreased, and Mg_2_Si was refined most clearly in Group S1. The thinner reaction region also implies a smaller volume of interfacial material available for continued reaction and eutectic growth, while Sc-related changes in local composition and solidification conditions can further modify the growth of γ-Al_12_Mg_17_ and Mg_2_Si. The final Sc-containing interface is therefore characterized by a reduced spatial reaction range and, depending on Sc content, a finer local solidification microstructure. Because no in situ solidification data were obtained, the observations are interpreted as restricted growth/coarsening rather than direct evidence of dynamic dendrite fragmentation.

The resulting mechanism can be expressed concisely as: Al-side Sc microalloying → modification of the Al-side microstructural/chemical state → confinement of interfacial reaction-zone expansion → modification of local solidification and second-phase growth → a thinner interface with fewer coarse γ-Al_12_Mg_17_ features and, at 0.6 wt.% Sc, refined Mg_2_Si. The following subsections examine the evidence for each step.

#### 4.2.1. Sc-Induced Suppression of Interfacial-Layer Growth

Growth of the Al/Mg reaction layer requires local heating and partial melting of the A356 surface, redistribution of Al, Mg, and Si, and continued formation/growth of Al_3_Mg_2_ and Al_12_Mg_17_. Since Sc was pre-introduced into the Al insert, its effect begins before the Al surface contacts the Mg melt. Studies of Sc-containing A356 and Al–Sc alloys show that Sc-related second phases can modify solidification microstructure and improve microstructural stability during thermal exposure [42,43,44,45,46,47,48,49,50,51,52,53]. In the present specimens, EDS detected discrete Al–Sc-rich and Mg-containing Sc-enriched regions; several Al–Sc-rich points were close to the nominal Al_3_Sc composition, but Al_3_Sc was not reliably indexed by EBSD. Accordingly, these features are treated as Sc-enriched or Al_3_Sc-related regions rather than as a fully confirmed continuous Al_3_Sc phase.

The key experimental constraint on the mechanism is that all Sc-containing specimens were thinner than Group A, whereas no continuous Sc-rich interlayer was observed. The total interface thickness decreased from 1750 μm to 1069, 1088, and 922 μm in S1, S2, and S3, respectively, and Group S1 also exhibited a narrower elemental transition range. A continuous physical blocking mechanism is therefore unlikely. A mechanism more consistent with the evidence is that the Sc-modified Al side changes the extent of local remelting/dissolution and the local solute supply to the reaction zone, thereby restricting the volume over which Al–Mg reaction phases and the subsequent eutectic region can continue to develop. This interpretation explains the simultaneous thinning of both the IMC and eutectic layers without requiring a change in the major reaction-phase system. Because diffusion coefficients, instantaneous dissolution rates, and interface-migration rates were not measured, the present data support confinement of the reaction range but do not prove that Sc directly reduces the intrinsic diffusion coefficient of Al or Mg.

#### 4.2.2. Refinement of the Interfacial Eutectic Microstructure by Sc

Sc also changed the morphology produced during interfacial solidification. Across S1–S3, coarse γ-Al_12_Mg_17_ dendritic features were reduced, with finer short-rod-like or locally discontinuous morphologies becoming more common; the reduction in coarse dendrites was especially evident in S2 and S3. In Al alloys, Sc-induced α-Al grain refinement is commonly attributed to heterogeneous nucleation on L1_2_-Al_3_Sc because of its favorable crystallographic matching with α-Al [44,46,47,48,54,55]. In the present work, however, the 23→19 μm statistic represents the equivalent circular diameter of indexed interfacial microstructural units rather than an isolated α-Al grain-size measurement; therefore, direct α-Al grain refinement is not claimed. For the γ-Al_12_Mg_17_ dendritic features, a mechanism consistent with the present observations is that the reduced reaction/eutectic-region volume decreases the space available for sustained dendrite growth, while Sc-related changes in local solute distribution and solidification conditions further limit growth and coarsening. Because the study contains only post-solidification images, the data support restricted dendritic growth and coarsening, not a directly observed fragmentation event.

#### 4.2.3. Regulation of the Mg_2_Si Phase by Sc

Mg_2_Si forms where Si supplied from the A356 side encounters Mg within the reacting interface. Group S1 showed the clearest Mg_2_Si response: its mean equivalent circular diameter decreased from 17 to 14 μm, its maximum size from 25 to 20 μm, and its size distribution became more concentrated. Elemental mapping also showed finer and more dispersed Si-enriched features. Studies of hypoeutectic Al–Mg–Si/Al–Mg_2_Si alloys have reported that Al_3_Sc can provide heterogeneous nucleation substrates for α-Al and Mg_2_Si eutectic, while Sc segregation and the associated constitutional undercooling ahead of the growing Mg_2_Si phase can suppress coarse Mg_2_Si growth [54,55]. These mechanisms provide a plausible literature-based explanation for the refinement observed in S1. However, because Al_3_Sc was not crystallographically confirmed in the present interface and no in situ solidification data were obtained, heterogeneous nucleation and growth-front segregation should be regarded as possible contributors rather than directly demonstrated mechanisms. Within the evidence available here, the Mg_2_Si refinement is most conservatively attributed to Sc-induced changes in local nucleation, solute distribution, and solidification conditions, together with the narrower reaction region, which limits the spatial extent available for subsequent Mg_2_Si growth and coarsening. At 0.9 and 1.2 wt.% Sc, blocky Sc-enriched regions became more frequent, and the Mg_2_Si refinement was weaker, demonstrating that the Mg_2_Si response to Sc was non-monotonic. The association suggests increasing local heterogeneity at higher nominal Sc additions, although a direct causal relationship between Sc-rich regions and Mg_2_Si growth requires further verification.

Taken together, Sc produces two related but non-identical interfacial responses. First, it confines growth of the overall reaction region, as shown by the reduced IMC, eutectic, and total interface thicknesses at all three Sc additions. Second, it modifies local solidification products, reducing coarse γ-Al_12_Mg_17_ and, most clearly at 0.6 wt.% Sc, refining Mg_2_Si and the overall microstructural scale. The absence of a continuous Sc-rich layer and the unchanged major reliably indexed phase types show that the mechanism is not a simple diffusion-barrier effect or phase-replacement mechanism. Instead, the evidence supports indirect regulation through the Sc-modified Al-side state, local solute distribution, and reaction/solidification conditions. Because S3 gave the thinnest interface whereas S1 gave the strongest Mg_2_Si refinement, no single Sc content can be defined as universally optimal from the present metrics alone.

### 4.3. Limitations and Future Work

The present study establishes a consistent relationship between Al-side Sc microalloying, interfacial thinning, and local microstructural refinement, but the proposed mechanism remains constrained by the available measurements. Diffusion coefficients, instantaneous Al-side dissolution rates, interface-migration rates, and in situ solidification behavior were not measured, so reaction-zone confinement should not be interpreted as direct proof of slower intrinsic diffusion. Al_3_Sc-related regions were inferred from EDS compositions and the literature rather than confirmed crystallographically by TEM/SAED, and detailed EBSD/mapping statistics were limited to Groups A and S1. In addition, hardness measurements were performed on one specimen per group with three spatially separated profiles and thus characterized within-specimen hardness distributions rather than specimen-to-specimen reproducibility; interfacial bonding strength and fracture behavior were not measured. Future work should combine quantitative composition verification of the Sc-modified inserts, high-resolution phase identification, diffusion/thermal analysis, in situ solidification characterization, and direct mechanical testing across all Sc levels to test the proposed mechanism and establish an application-specific optimum Sc content. The EBSD size statistics were based on one analyzed phase map for Group A and one for Group S1, with a scan step size of 3.5 μm. The limited spatial sampling and field coverage constrain the precision and representativeness of small-feature size measurements.

## 5. Conclusions

A356/AZ91D bimetallic composites with nominal Sc additions of 0–1.2 wt.% in the Al insert were fabricated by lost foam compound casting. The results show that Al-side Sc microalloying primarily regulates the spatial extent and growth morphology of the existing Al/Mg reaction/solidification structure rather than replacing the major interfacial phase system. The main conclusions are as follows:

(1) Sc addition markedly reduced the interfacial reaction range. The total interface thickness decreased from 1750 μm in Group A to 1069, 1088, and 922 μm in S1, S2, and S3, corresponding to reductions of 39%, 38%, and 47%, respectively. Both the IMC and eutectic layers became thinner. The response was non-monotonic, with S3 showing the minimum total thickness.

(2) Sc also modified the local solidification microstructure. In S1, the mean equivalent circular diameter of indexed interfacial microstructural units decreased from approximately 23 to 19 μm, while the mean and maximum Mg_2_Si sizes decreased from approximately 17 to 14 μm and from 25 to 20 μm, respectively. Coarse γ-Al_12_Mg_17_ dendritic features were reduced across all Sc-containing groups. At higher Sc additions, blocky Sc-enriched regions became more frequent and Mg_2_Si refinement weakened, showing that refinement was also non-monotonic.

(3) Sc did not produce a marked change in the major reliably indexed interfacial phases or in the overall preferred orientation, and elemental mapping showed discrete Sc enrichment rather than a continuous Sc-rich interlayer. These observations rule out a simple continuous-barrier or phase-replacement explanation. The combined SEM/EDS, EBSD, mapping, and size-statistics evidence is instead consistent with an indirect mechanism in which Sc first modifies the Al-side microstructural/chemical state, then confines expansion of the interfacial reaction region and alters local solidification, thereby restricting growth/coarsening of γ-Al_12_Mg_17_ and Mg_2_Si. For Mg_2_Si, the literature on Sc-containing Al–Mg–Si alloys supports altered heterogeneous nucleation and growth-front solute conditions as plausible contributors, whereas the γ-Al_12_Mg_17_ change is interpreted more conservatively as restricted dendritic growth within the confined reaction/eutectic region. These mechanistic interpretations describe the observed reaction range and morphology but should not be taken as direct measurements of atomic diffusivity, nucleation kinetics, or in situ growth behavior.

(4) The high-hardness interfacial region narrowed in parallel with the thinner interface, while locally high hardness was retained. This change reflects the reduced spatial extent of hard Al–Mg reaction products rather than a demonstrated increase in interfacial bonding strength. Because S3 produced the thinnest interface whereas S1 produced the strongest Mg_2_Si refinement, the preferred Sc addition depends on the target interfacial characteristic; a universal optimum cannot be assigned from thickness and refinement alone.

## Figures and Tables

**Table 1:** Chemical compositions of the as-cast A356 and AZ91D alloy ingots (wt.%).

	Al	Mg	Zn	Mn	Si	Fe	Ti
A356	Bal.	0.34	-	-	7.56	0.10	0.11
AZ91D	8.66	Bal.	0.61	0.24	0.02	-	-

**Table 2:** EDS point-analysis results for the locations marked in Figure 2b–d (wt.%).

Point	Element						Phase
	Mg	Al	Si	Ti	Mn	Fe	
1	64.36	-	35.64	-	-	-	Mg_2_Si
2	35.95	64.05	-	-	-	-	Al_3_Mg_2_
3	54.96	45.04	-	-	-	-	Al_12_Mg_17_
4	54.13	45.87	-	-	-	-	Al_12_Mg_17_
5	53.96	46.06	-	-	-	-	Al_12_Mg_17_
6	63.21	-	36.79	-	-	-	Mg_2_Si
7	70.19	29.81	-	-	-	-	δ-Mg
8	57.88	42.12	-	-	-	-	Al_12_Mg_17_
9	76.22	23.78	-	-	-	-	δ-Mg

**Table 3:** EDS point-analysis results for the locations marked in the Sc-containing interface micrographs (wt.%).

Point	Element						Phase/Region
	Mg	Al	Si	Ti	Mn	Sc	
1	60.62	-	39.38	-	-	-	Mg_2_Si
2	34.96	65.04	-	-	-	-	Al_3_Mg_2_
3	53.59	46.41	-	-	-	-	Al_12_Mg_17_
4	-	68.50	-	-	-	31.50	Al_3_Sc-related phase
5	-	71.59	-	-	-	28.41	Al_3_Sc-related phase
6	57.33	42.67	-	-	-	-	Al_12_Mg_17_
7	63.16	6.36	30.48	-	-	-	Mg_2_Si
8	30.50	55.93	-	-	-	13.56	Mg-containing Sc-enriched region
9	23.06	58.37	-	-	-	18.57	Mg-containing Sc-enriched region
10	54.33	45.67	-	-	-	-	Al_12_Mg_17_
11	79.36	20.64	-	-	-	-	δ-Mg
12	15.56	66.40	-	-	-	18.04	Mg-containing Sc-enriched region
13	22.57	66.00	-	-	-	11.43	Mg-containing Sc-enriched region
14	-	66.13	-	-	-	33.87	Al_3_Sc-related phase
15	22.17	60.76	-	-	-	17.08	Mg-containing Sc-enriched region
16	9.96	60.71	-	-	-	29.33	Mg-containing Sc-enriched region
17	17.17	62.34	-	-	-	20.49	Mg-containing Sc-enriched region

## Data Availability

The original contributions presented in this study are included in the article. Further inquiries can be directed to the corresponding author.
